# Molecular Cloning and Characterization of PnbHLH1 Transcription Factor in *Panax notoginseng*

**DOI:** 10.3390/molecules22081268

**Published:** 2017-07-29

**Authors:** Xiang Zhang, Feng Ge, Bing Deng, Taif Shah, Zhuangjia Huang, Diqiu Liu, Chaoyin Chen

**Affiliations:** Faculty of Life Science and Technology, Kunming University of Science and Technology, Kunming 650500, China; 18213578169@163.com (X.Z.); iogoing@163.com (B.D.); taifshah@163.com (T.S.); cdcdheifeng@163.com (Z.H.); dianaloveyou2006@163.com (D.L.); aqjingsheng@163.com (C.C.)

**Keywords:** *Panax*, transcription factors, triterpene, saponin, over-expression

## Abstract

*Panax notoginseng* has been extensively used as a traditional Chinese medicine. In the current study, molecular cloning and characterization of PnbHLH1 transcription factor were explored in *Panax notoginseng*. The full length of the *PnbHLH1* gene obtained by splicing was 1430 bp, encoding 321 amino acids. Prokaryotic expression vector *pET-28a-PnbHLH1* was constructed and transferred into the *BL21* prokaryotic expression strain. An electrophoretic mobility shift assay of PnbHLH1 protein binding to E-box cis-acting elements verified that PnbHLH1 belonged to the bHLH class transcription factor which could interact with the promoter region of the E-box core sequence. The expression levels of key genes involved in the biosynthesis of triterpenoid saponins in *PnbHLH1* transgenic cells were higher than those in the wild cells. Similarly, the total saponin contents were increased in the *PnbHLH1* transgenic cell lines compared with the wild cell lines. Such results suggest that the *PnbHLH1* transcription factor is a positive regulator in the biosynthesis of triterpenoid saponins in *Panax notoginseng*.

## 1. Introduction

**Figure 1:**
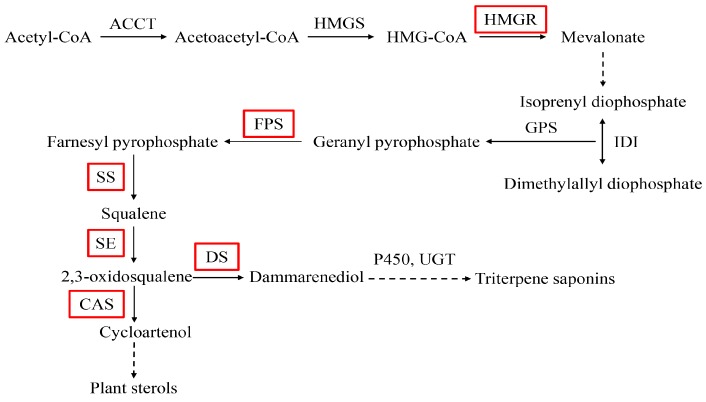
Biosynthetic pathway of triterpene saponins in *P. notoginseng*. AATC: acetoacetyl-CoA acyltransferase; HMGS: 3-Hydroxy-3-methylglutaryl-CoA synthase; HMGR: 3-hydroxy-3-methylglutaryl-CoA reductase; IDI: isopentenyl diphosphate isomerase; GPS: geranylgeranyl pyrophosphate synthase; FPS: farnesyl diphosphate synthase; SS: squalene synthase; SE: squalene epoxidase; DS: dammarenediol-II synthase; P450: cytochrome P450; UGP: UDP giycosyltransferase; CAS: cycloartenol synthase. The dotted lines indicate several enzyme reactions. The red boxes represent the key enzymes.

*Panax notoginseng* (Burk) F. H. Chen is a highly-valued medicinal plant, belonging to the *Panax* genus of the Araliaceae family. The main bioactive components are saponins which are used in the treatment of cardiovascular diseases, nervous system diseases, anti-inflammation, and anti-aging [1]. *P. notoginseng* saponins (PNS) are triterpenoids mainly synthesized through the mevalonic acid (MVA) pathway [2] (Figure 1).

Transcription factors are DNA binding proteins which bind to the promoter region of genes to regulate gene expression [3]. The operating transcription factor is an efficient strategy to control the biosynthetic pathway because of its unique multi-point regulation. In recent years, with the elucidation of the secondary metabolic pathway of plants and regulatory mechanisms, especially the identification of transcription factors regulating the biosynthesis of specific secondary metabolites, gene engineering based on transcription factors has been proven to have strong effects on improving the yields of target metabolites [4]. The basic-helix-loop-helix (bHLH) type transcription factor domain is composed of about 60 amino acids, including the basic amino acid region and α-helix-ring-α-helix region that plays important roles in the development of plant metabolism [5]. The basic amino acid region is located at the N-terminus of the bHLH transcription factor which has a binding site and can identify DNA [6].

Terpenoids are pharmacological ingredients mainly found in plants in low amounts, so their price is extremely high. The research on the terpenoid biosynthetic pathway has become a hotspot in the field of metabolic engineering [7]. Transcriptional activation of transcription factor is an important regulatory link in secondary metabolite biosynthesis. As an elicitor, methyl jasmonate (MeJA) did not directly affect the synthesis of terpenoids, however, it could induce the expressions of some transcription factors which control the synthesis of terpenoids in *P. notoginseng* [8]. Research showed that when the transcription factor gene (*PqWRKY1*) was over-expressed in *P. quinquifolium*, the expression levels of key enzyme genes involved in the pathway of triterpenoid saponin’s biosynthesis in transgenic cells were 1–5 times higher than those in non-transgenic cell lines [9]. In addition, the binding of transcription factors with DNA in vitro helped to elucidate the regulation and improve the yield of PNS in *P. notoginseng*. In this study, the interaction between the PnbHLH1 transcription factor and the E-box cis-element was verified by an electrophoretic mobility shift assay (EMSA) and the *PnbHLH1* transcription factor gene was transferred into *P. notoginseng* cells to carry out functional analysis. Furthermore, the molecular cloning and characterization of the PnbHLH1 transcription factor in *P. notoginseng* provided a theoretical reference and basis for obtaining a stable and efficient regulation of the PNS biosynthesis.

## 2. Results and Discussion

### 2.1. Cloning of PnbHLH1 Gene

The PnbHLH1 transcription factor was obtained by the yeast one-hybrid system. After sequencing, the full length of the *PnbHLH1* gene was 1430 bp, consisted of a 5′ untranslated region, a 3′ untranslated region, a poly (A) tail, and an open reading frame (ORF). Similarly, 966 bp encoding 321 amino acids with 36.1 kD molecular weight and 6.97 isoelectric points were recorded.

According to the reports, the JERE (JA- and elicitor-responsive element, CTCTTAGACCGCCTTCTTTGAAAG) sequence binds to the relevant transcription factors which are related to the biosynthesis of terpenoids [8]. We inserted the JERE element into the multiple cloning site of the bait plasmid that was specifically designed for the yeast one-hybrid system and used to identify DNA-binding proteins, and screened for *PnbHLH1* which might relate to the biosynthesis of the terpenoids in *P. notoginseng*.

### 2.2. Bioinformatics Analysis of PnbHLH1 Transcription Factor

In the coding region of *PnbHLH1* (140-206 AA), bHLH binding domain was formed by 67 amino acids, which had high homology with the binding domain of other bHLH transcription factors (TFs) [6], indicating that PnbHLH1 might belong to the bHLH class TF. The protein sequence encoded by the *PnbHLH1* gene was analyzed by SignalP 4.1 Server and it was found that the protein N-terminal had no leader peptide, indicating that the protein was not a secreted protein. According to the PredictProtein online tool, this protein might be located in the nucleus.

**Figure 2:**
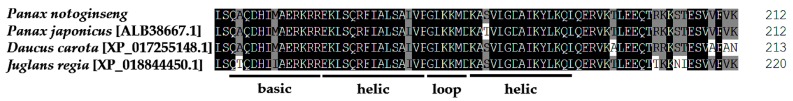
Alignment of deduced amino acid sequences of PnbHLH1 with the proteins of other plants bHLH. The domain of the bHLH class transcription factor was signed with horizontal lines (gray shading indicates the identity level > 75%, black shading indicates the identity level = 100%).

**Figure 3:**
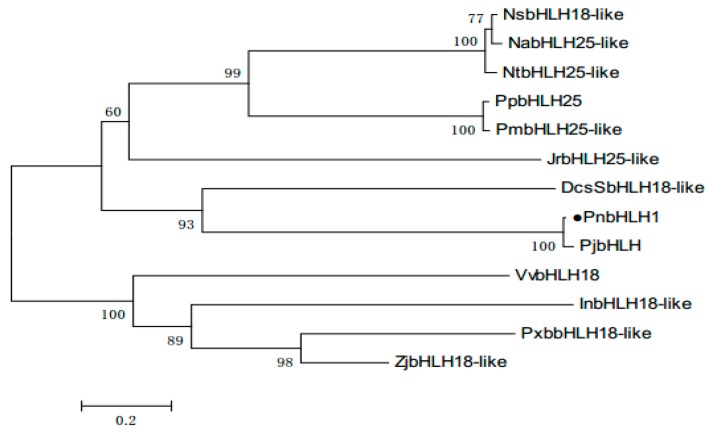
Phylogenetic analysis of PnbHLH1 protein sequences with other bHLH proteins. The sequences used in this experiments were NsbHLH18-like (*Nicotiana sylvestris*, XP_009779237.1), NabHLH25-like (*Nicotiana attenuate*, XP_019232955.1), NtbHLH25-like (*Nicotiana tomentosiformis*, XP_009616821.1), PpbHLH25 (*Prunus persica*, XP_00720035 1.1), PmbHLH25-like (*Prunus mume*, XP_008236173.1), JrbHLH25-like (*Juglans regia*, XP_018844450.1), DcsSbHLH18-like (*Aucus cariota subsp. Sativus*, XP_017255148.1), PjbHLH (*Panax japonicas*, ALB38667.1), VvbHLH18 (*Vitis vnifera*, XP_002268443.3), InbHLH18-like (*Ipomoea nil*, XP_019169484.1), PxbbHLH18-like (*Pyrus bretschneideri*, XP_009346451.1), and ZjbHLH18-like (*Ziziphus jujube*, XP_015893295.1).

With the help of BLASTP, we found that PnbHLH1 has high homology with known bHLH TFs in many plants, PnbHLH1 shared 97% sequence identities with *Panax japonicus* bHLH (ALB38667.1), 73% with *Daucus carota* bHLH18-like (XP_017255148.1), and 62% with *Juglans regia* bHLH25-like (XP_018844450.1), but the function of these similar TFs is not determined (Figure 2). Like other plant bHLH proteins, PnbHLH also contained the basic amino acid region and the α-helix-ring-α-helix region. The neighbor-joining method phylogenetic tree was constructed using the bootstrap method of MEGA 6.0 with 1000 replications and the respective plant species of the above proteins are shown in the tree (Figure 3). The black circular symbols indicated the PnbHLH1 protein. The structure and functions of the PnbHLH1 TF could be predicted to a certain extent by constructing the phylogenetic tree with 12 TFs which confirmed that the amino acid sequence isolated in this report was a bHLH TF gene of *P. notoginseng*.

**Figure 4:**
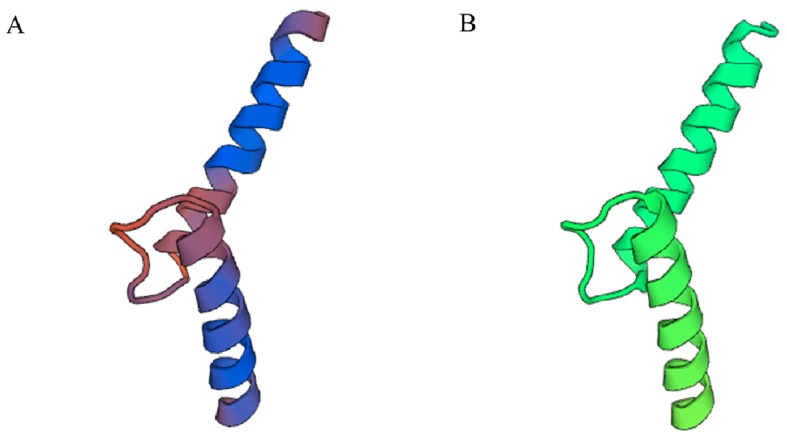
Three-dimensional structure prediction of PnbHLH1 (**A**) and MYC2 (**B**) proteins.

The 3D structure of PnbHLH1 was constructed with the SwissModel to clarify the structure–function relationship of plant bHLH TFs. Using the crystal structure of the transcription factor MYC2(5gnj.4.B) as a template, the 3D model of PnbHLH1 was built and shared 62.00% identity with the TF (Figure 4).

According to the reports, bHLH TFs in *Arabidopsis* are abundant [10], similarly, CrMYC belongs to the bHLH class TFs in *Catharanthus roseus* [11]. Moreover, CrMYC1 and CrMYC2 could regulate the expressions of some key genes involved in the biosynthesis of terpenoids in *C. roseus*. Based on the previous studies, PnbHLH1 might be involved in the biosynthesis of terpenoids in *P. notoginseng*, which should be studied next.

### 2.3. Electrophoretic Mobility Shift Assay Experiment of PnbHLH1 and Cis-Acting Element E-Box

**Figure 5:**
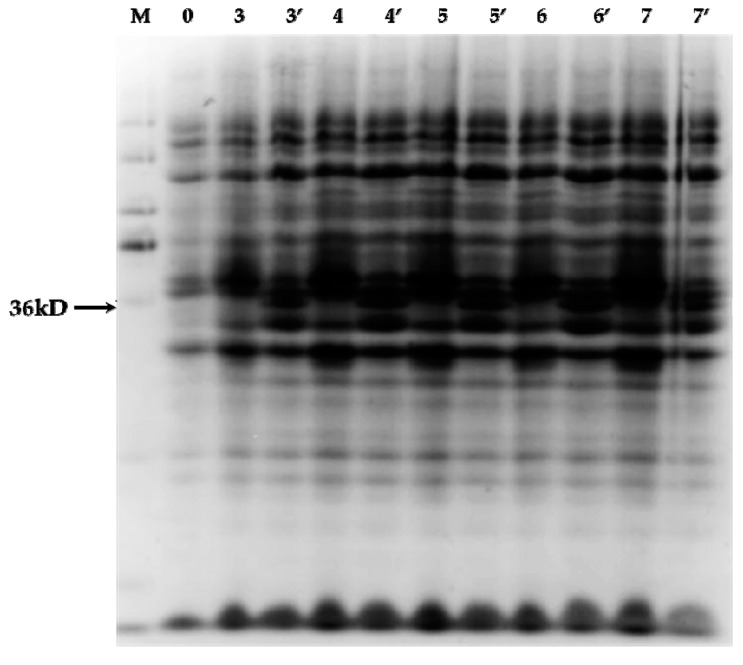
Expression of His-PnbHLH1 proteins. M: Marker ruler (120, 100, 80, 60, 50, 40, 30, 20, 12 kD); 0: total proteins of pET-28a-PnbHLH1 before induction; 3, 4, 5, 6, and 7: total proteins of pET-28a-PnbHLH1 were induced at 3, 4, 5, 6, and 7 h without IPTG respectively; 3′, 4′, 5′, 6′, and 7′: total proteins of pET-28a-PnbHLH1 were induced at 3, 4, 5, 6, and 7 h by IPTG, respectively.

Electrophoretic mobility shift assay (EMSA) in vitro can objectively and effectively reflect the potential of the interaction between the target protein and nucleic acid. *Bacteriophage DE3*-solubilized *BL21* (*DE3*)-specific strains were engineered on the basis of the *BL21* strain for integrating the T7 RNA polymerase gene into the bacterial genome. The recombinant *pET-28a-PnbHLH1* vector was transformed into *BL21* (*DE3*) for expression. After removal of the labeled protein, the size of the band was consistent with the predicted molecular weight of the PnbHLH1 protein which was 36 kD, indicating that the *pET-28a-PnbHLH1* recombinant plasmid was stably expressed when the induction treatment was carried out at 4 h and 5 h by isopropy-β-d-thiogalactoside (IPTG) in the *BL21* (*DE3*) strain (Figure 5).

In many cases, the exogenous proteins were aggregated into insoluble inclusion bodies that were biologically non-active [12]. His-PnbHLH1 was expressed in the form of an inclusion body, then dissolved under denaturing conditions and purified by affinity [13]. Dialysis was used to recover the spatial structure and biological activity of His-PnbHLH1 protein. Due to a small amount of precipitation in the refolding process, the protein concentration decreased and needed to be concentrated. The concentration of His-PnbHLH1 protein had been greatly improved by a Millipore ultrafiltration tube.

**Figure 6:**
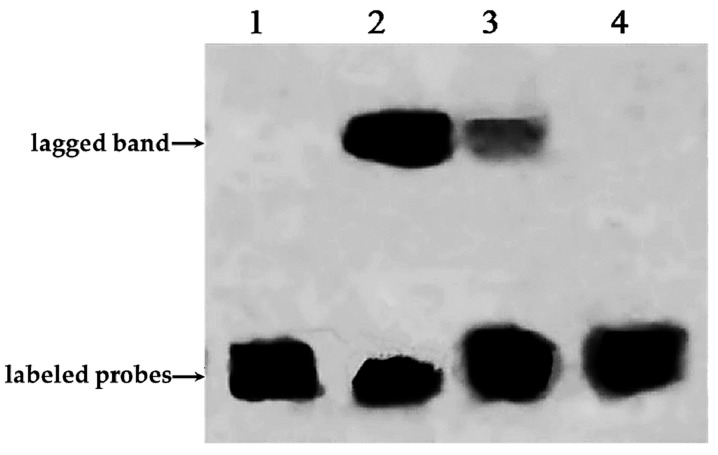
Electrophoretic mobility shift assay results of PnbHLH1 protein binding to E-box cis-acting elements. 1. Adding free probes labeled with biotin; 2. Adding free probes labeled with biotin and target protein; 3. Adding free probes labeled with biotin, target proteins, and competitive probes not labeled with biotin; and 4. Adding mutant probes labeled with biotin and target protein.

E-box (5′-CANNTG-3′) is a transcription factor binding site and recognized by proteins that can help initiate transcription of the gene. The CA nucleotides of the hexanucleotide sequence can be directly contacted with a glutamic acid (E) residue at position 9 of all E-box–binding bHLH proteins [6]. The purified His-PnbHLH1 fusion protein incubated with free probes (containing the E-box core sequences labeled with biotin), competitive probes (containing the E-box core sequences but being not labeled with biotin), and mutant probes (containing mutations in core sequences labeled with biotin), respectively. The results of EMSA were shown in Figure 6. In contrast to lane 1 and lane 2, it was found that a lagged band appeared clearly after adding the PnbHLH1 target protein. Competitive probes which were 50 times that of the free probe on the basis of lane 2, were added in lane 3. These competitive probes can be fully combined with the PnbHLH1 protein, but the probes were not labeled with biotin and could not be chromogenic. As shown in Figure 6 (lane 3), competitive probes attached to most of the PnbHLH1 protein; therefore, only a small amount of free probes could combine with the protein and produce a light lagged band compared with that in lane 2. In the lane 4, the mutant probes labeled with biotin could not interact with the PnbHLH1 protein, and there was no lagged band. 

All the results indicated that PnbHLH1 TF could bind with the E-box cis-acting element specifically. Most of the bHLH transcription factors could recognize E-box [14], so PnbHLH1 protein may belong to bHLH TFs. Similarly, we have demonstrated that the promoters of key genes (*DS*, *SS*, and *SE*) involved in PNS biosynthesis in *P. notoginseng* contained E-box core site sequences. Such results showed that PnbHLH1 TF might interact with the E-box cis-elements in these promoters to regulate the expressions of such genes, which would be proven in the next experiment.

### 2.4. Expression Analysis of PnbHLH1 and Other Key Enzyme Genes in Transgenic Cells

Ginsenosides have been obtained from the cells in mass quantity [15]. With the rapid development of biotechnology and bioinformatics, numerous studies based on molecular levels have been carried out in *Panax* species, such as *P. ginseng* and *P. quinquefolius*, in order to improve the yield of the plant itself, or the active ingredient. In this study, genetic engineering and metabolic engineering were applied to investigate the regulation of PNS biosynthesis in *P. notoginseng* cells.

**Figure 7:**
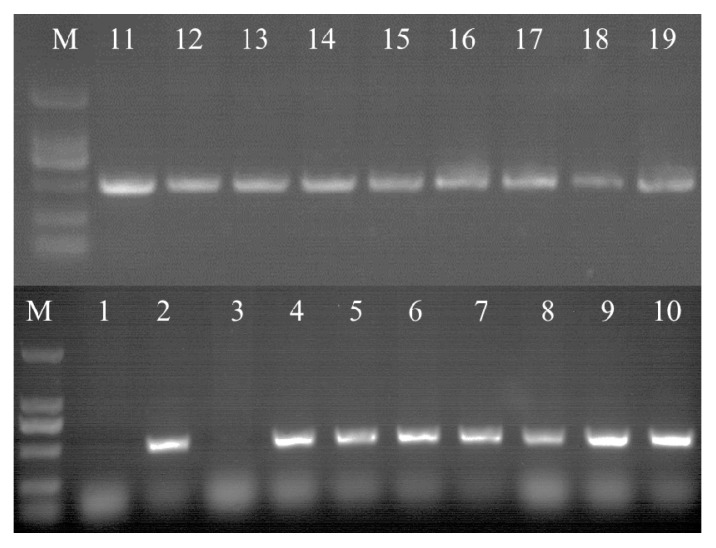
PCR analysis of *HPT* (500 bp) in transgenic cell lines. M: DNA Marker DL 2000. 1: negative control (non-transgenic cell line); 2: positive control (vector carrying the *HPT* gene); and 3–19: transgenic cell lines.

In order to obtain *PnbHLH1* over-expressed cell lines, the recombinant *pCAMBIA1300s-PnbHLH1* vector was transferred into *P. notoginseng* cells and 16 anti-Hyg cell lines were generated. Genomic DNA of transgenic cells was extracted by the CTAB method, and the integrity was checked by agarose gel electrophoresis. Using DNA as a template, specific primers were designed according to the hygromycin resistance gene (*HPT*) on T-DNA for screening the positive transgenic cells by PCR. The positive rate of *HPT* was 90% of the transgenic cell lines (Figure 7).

**Figure 8:**
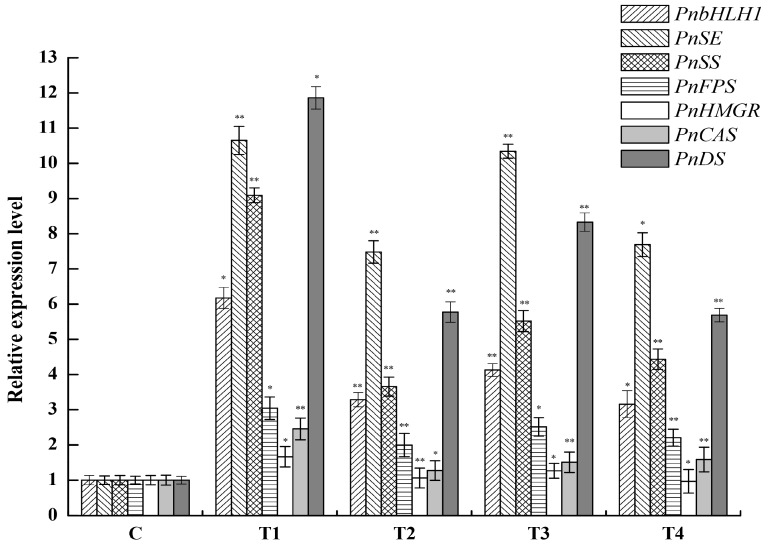
Relative expression levels of *PnbHLH1*, *PnDS*, *PnSS*, *PnSE*, *PnHMGR*, *PnFPS*, and *PnCAS* in four *PnbHLH1*-transgenic *P. notoginseng* cell lines (*, *p* < 0.05; **, *p* < 0.01). *18s rRNA* was selected as the internal reference gene.

Four cell lines which grew well were selected to explore the expression levels of *PnbHLH1*, *PnDS*, *PnSS*, *PnSE*, *PnHMGR*, *PnFPS*, and *PnCAS* involved in the biosynthesis of triterpene saponins in transgenic cells using qRT-PCR. *PnbHLH1* was successfully over-expressed in the four transgenic cells lines; meanwhile, the expression levels of *PnDS*, *PnSS*, *PnSE*, and *PnFPS* genes were also significantly increased compared with those in normal *P. notoginseng* cells (control cells) (Figure 8).

Nevertheless, the expression level of *PnHMGR* did not change much and such a gene might be one of the key factors in modulating the biosynthesis of triterpenoid saponins [16]. *PnCAS* expression level was slightly higher than that in control. Moreover, as the promoters of key genes (*DS*, *SS*, and *SE*) involved in PNS biosynthesis contained E-box core site sequences which could interact with PnbHLH1 TF in theory, the expression levels of *DS*, *SS*, and *SE* should be influenced in *PnbHLH1* over-expressed cells. The qRT-PCR assays showed that over-expression of the *PnbHLH1* TF gene in *P. notoginseng* cells could exactly increase the expression levels of *DS*, *SS*, and *SE* significantly, suggesting that the PnbHLH1 TF could interact with some key enzyme genes and regulate the expressions of them. Such results represented that TFs had multi-point regulations in secondary metabolic pathways and the biosynthesis of saponins in *P. notoginseng* cells might be strengthened.

### 2.5 Analysis of Saponins in Transgenic Cells

**Figure 9:**
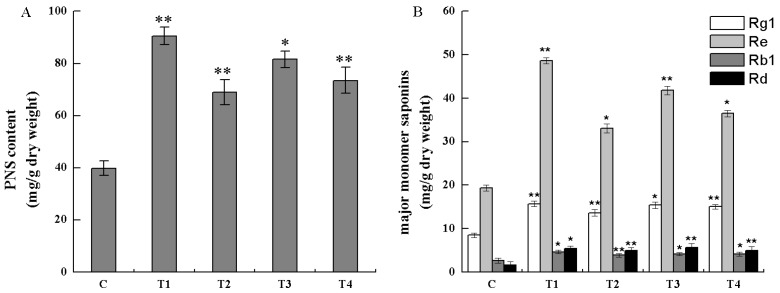
Analysis of saponins in the cell lines (*, *p* < 0.05; **, *p* < 0.01).

Total saponins of the transgenic cell lines (T1, T2, T3, and T4) and the non-transgenic cells (control cell) were extracted and analyzed by spectrophotometer absorbance. As shown in Figure 9A, the contents of total saponins in T1, T2, T3, and T4 cell lines were 2.27, 1.73, 2.05, and 1.85 times of those in non-transgenic cell line, respectively, which indicated that *PnbHLH1* over-expressed in cells could significantly enhance the biosynthesis of PNS. PnbHLH1 transcription factor did not regulate PNS biosynthesis directly, however, it was able to interact with key enzyme genes involved in the PNS biosynthetic pathway and, as a result of this, PnbHLH1 increased the PNS biosynthesis by means of augmenting the expression levels of several key genes in the PNS biosynthesis pathway simultaneously.

Similarly, four kinds of monomer saponins (Rg1, Re, Rb1, and Rd) in cells were determined by HPLC, which were the main monomer saponins in *P. notoginseng* (Figure 9B). The content of four monomer saponins in transgenic cell lines (T1, T2, T3, and T4) were all increased significantly in comparison to non-transgenic cell lines. Among the four monomer saponins, Re had the highest content in cells, in which biosynthesis was also strengthened to the greatest extent in transgenic cells. The average content of Re was about 40.01 mg/g, especially in the T1 cell line, Re content was up to 48.64 mg/g. The average Rg1 content was 1.76 times that found in non-transgenic cells and the highest content was presented in the T3 cell line. The contents of Rd in the *PnbHLH1* transgenic gene lines T1, T2, T3, and T4 were 3.88, 2.73, 3.39, and 3.03 times that found in non-transgenic cells, respectively. Rd was a small polar saponin and its pharmacological activity was stronger than Rb1, but the content of Rd was lower in *P. notoginseng* compared with Rb1. In order to enhance the production of Rd, our previous study has shown that *Trichoderma longibrachiatum* could especially convert Rb1 to Rd [17]. In this research, it was found that the content of Rb1 in the non-transgenic cell lines was higher than Rd, while the content of Rd in four transgenic cell lines were all higher than Rb1, which suggested that over-expression of *PnbHLH1* might activate the special Rd synthetic pathway, especially the complicated modification of saponin framework.

Transcription factors are used as an efficient tool for the activation and transcription of genes in plant metabolic pathways with its unique ‘multi-point regulation’ advantage, which can regulate the expressions of several genes in the meantime and have become a new strategy to make up the deficiency of metabolic engineering targeting one or two key enzyme genes. We have regulated the expressions of two key genes involved in the biosynthetic pathway of *P. notoginseng* saponins in a previous study [18], which increased the biosynthesis of saponins. On the basis of the former study [18], it has been demonstrated that manipulation of the transcription factor was more efficient than controlling key enzyme genes in the promotion of PNS biosynthesis. For affecting multiple gene expressions in plant secondary metabolic pathways, identification, characterization, and application of TFs which related to secondary metabolite biosynthesis are an interesting and effective strategy [19].

## 3. Materials and Methods

### 3.1. Plant Materials

In this study, cells of *P. notoginseng* were cultured on Murashige and Skoog (MS) agar medium supplemented with 2.0 mg L^−1^ 2, 4-dichlorophenoxyacetic acid and 1.0 mg L^−1^ kinetin, pH 5.8 in the dark at 25 °C for four weeks.

### 3.2. Gene Sequencing, Phylogenetic Gene Structure, and Conserved Motif Analyses

Partial fragments of genes were obtained by Matchmaker™ Gold Yeast One-Hybrid Library Screening System (Clontech, Mountain View, CA, USA). Similarly, the full length of *PnbHLH1* was obtained by a Smarter™ Race cDNA amplification kit (Clontech, USA). The bHLH1 amino acid sequence of *P. notoginseng* was aligned with Interpro (http://www.ebi.ac.uk/interpro/). The PnbHLH1 protein sequence was analyzed by SignalP 4.1 Server (http://www.cbs.dtu.dk/services/SignalP/) and the subcellular location of PnbHLH1 protein was predicted by PredictProtein (https://www.predictprotein.org/). The phylogenetic tree was constructed by MEGA 5.0 and the three-dimensional structure of PnbHLH1 was predicted by Swiss-Model (https://swissmodel.expasy.org/).

### 3.3. Constructions of Plant Expression Vector and Prokaryotic Expression Vector Carrying the PnbHLH1 Gene

**Figure 10:**
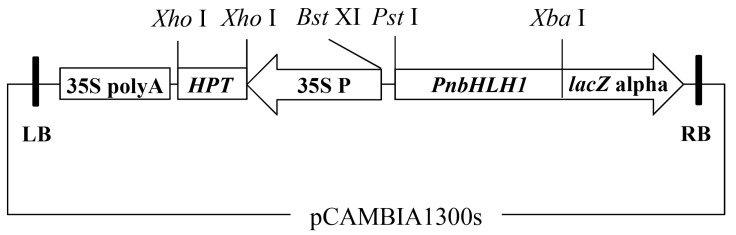
T-DNA region of the plant expression vector pCAMBIA1300s-*PnbHLH1*. Intermediates: 35S poly A, terminator of *CaMv 35S* gene; *HPT*, hygromycin phosphotransferase gene; 35S P, 35S promoter.

The plant expression vector harboring the *PnbHLH1* gene was constructed as described previously [20]. Briefly, double digestions of plasmids *pGEM-T-PnbHLH1* and *pCAMBIA1300S* by *Pst* I and *Xba* I were conducted to isolate the fragments of *PnbHLH1* and linear *pCAMBIA1300S*. These two DNA fragments were then ligated to form the recombinant binary plant expression vector *pCAMBIA1300S-PnbHLH1* (Figure 10). This recombinant vector was sequenced to ensure there were no wrong DNA fragments in the target gene. The open reading frame (ORF) of *PnbHLH1* was cloned into *pET-28a* prokaryotic expression vector using *Sal* I and *Nde* I restriction enzymes.

### 3.4. Transformation of the PnbHLH1 Plant Expression Vector Mediated by Agrobacterium

The expression vector *pCAMBIA1300S*-*PnbHLH1* was transformed into *A*. *tumefaciens* (*EHA105*) using the freeze–thaw method [21]. Turbid bacterial solution (1.0 mL) was aspirated onto Luria-Bertani (LB) agar medium containing 50.0 mg/L kanamycin sulfate (Kan) and 25.0 mg/L rifampicin (Rif) at 28 °C for 72 h. Then the *A*. *tumefaciens* was moved into MGL liquid medium (Tryptone 5.0 g/L, NaCl 5.0 g/L, Mannitol 5.0 g/L, MgSO_4_·7H_2_O 0.1 g/L, KH_2_PO_4_ 0.25 g/L, and Glycine 1.0 g/L, pH 5.8) which was added to 40.0 mg/L acetosyringone. The strain culture was stopped when the OD_600_ reached 0.6. The fresh wild-type (WT) cells of *P. notoginseng* were transferred to MS medium supplemented with 40.0 mg/L acetosyringone for three days in advance and completely immersed in the *A*. *tumefaciens* liquid for shaking.

### 3.5. Identification of Transgenic Cells by PCR

Genomic DNA was extracted by the cetyltrimethylammonium bromide (CTAB) method and used for the identification of *HPT* II based on Ex Taq^®^ (Takara, Dalian, China) in the transgenic cells. The primers were: Hpt II-F (5′-GAAGTGCTTGACATTGGGGAAT-3′) and Hpt II-R (5′-AGATGTTGGCGACCTCGTATT-3′). A 25.0 μL solution was used for the PCR reaction system containing 0.25 μL Ex Taq^®^, 2.0 μL of dNTP mixture (2.5 mM), 2.5 μL of 10×Ex Taq Buffer, 0.25 μL of each of the forward and reverse primers (10 μM), 2.0 μL of DNA template, and 17.75 μL of ddH_2_O. The PCR reaction conditions were as follows: 95 °C for 5 min, 95 °C for 30 s, 55 °C for 30 s, 72 °C for 30 s for an optimal number of 32 cycles, and 72 °C for 10 min.

### 3.6. Expression Analysis by qRT-PCR

Total RNA was isolated from each sample by the guanidine thiocyanate procedure [22]. The concentration and quality of RNA were measured using agarose gel electrophoresis and an Ultrospec 2100 pro spectrophotometer (GE, Fairfield, CT, USA). The first cDNA strand was synthesized using the GoTaq^TM^ Reverse Transcription System (Promega, Madison, WI, USA). The specific primers were designed by Primer Premier 5.0, as previously described [23]. *18S rRNA* served as the reference gene. The quantitative reaction was conducted using GoTaq^®^ qPCR Master Mix Real-Time PCR System (Promega, USA) for 40 cycles with the following reaction conditions: 2 min at 95 °C, followed by 40 cycles of 95 °C for 15 s, 55 °C for 30 s, and 72 °C for 30 s (96, Roche, Swiss). The expression levels of *PnCAS* (cycloartenol synthase), *PnDS* (dammarenediol synthase), *PnHMGR* (3-hydroxy-3-methyl-glutaryl coenzyme A reductase), *PnFPS* (farnesyl pyrophosphate), *PnSS* (squalene synthase), *PnSE* (squalene epoxidase), and *PnbHLH1* were analyzed (Table 1).

### 3.7. Analysis of Triterpenoid Saponins

Cells were collected and dried to a constant weight at 55 °C, and then thoroughly ground into powder. A small amount of each sample (0.50 g) was transferred into a centrifuge tube containing 20.0 mL methanol, followed by ultrasonic treatment for 2 h. An HPD-100 macroporous resin column was used for loading the methanol extracts. The extracts of different transgenic cells and non-transgenic cells were dried in an oven at 50 °C and diluted with 70% methanol to 25.0 mL. The absorbance was measured according to the methods of the A_550_ and the standard curve and PNS in each sample was calculated. The systems and samples used in it are as described in our previous study [24]. Monomer saponin content was determined by the high-performance liquid chromatography (HPLC) on an ULTIMATE 3000 LPG-3400A system (Dionex, Sunnyvale, CA, USA) using the 25.0 mL extracts. A Waters Symmetry C18 column (5 µm, 4.6 × 250 mm) was used for the HPLC analysis with water and acetonitrile as the mobile phase. The retention time and peak areas were performed to identify and quantify the ginsenosides calculated with standard ginsenosides purchased from the National Institutes for Food and Drug Control (Beijing, China).

### 3.8. Analysis of PnbHLH1 Binding with DNA

Prokaryotic expression vector was transformed into *BL21* (*DE3*)-competent cells and the positive monoclonal cell was inoculated in LB liquid medium (containing 50.0 mg/L Kan) incubated overnight at 37 °C. The bacterial liquid was inoculated into LB liquid medium with a dose of 1% at 37 °C until OD_600_ reached 0.8. The protein was induced by the addition of isopropyl β-d-1-thiogalactopyranoside (IPTG) to the final concentration of 0.4 mM. The bacterial fluids at 3, 4, 5, 6, and 7 h were collected and centrifuged for 2 min; the precipitate was suspended with 1 × phosphate-buffered saline and detected by sodium dodecyl sulfate polyacrylamide gel electrophoresis (SDS-PAGE). The target protein was purified from the cells by using His-tag protein purification kit. The probe sequences containing E-box core sequences and mutations in core sequences were artificially synthesized (Table 2), which were incubated for 5 min at 98 °C and slowly cooled to form double-stranded probes. The purified *PnbHLH1* protein was incubated with the probes at room temperature according to the standard procedure of the EMSA kit (PIERCE, Holmdel, NJ, USA).

### 3.9. Statistical Analysis

All the data were derived from three separate replicates. Values were represented as the mean ± SD, which was calculated from three replicates. Significant differences based on Student’s *t*-test in measured parameters were indicated with asterisks (*, *p* < 0.05; **, *p* < 0.01).

## 4. Conclusions

In this study, a transcription factor, *PnbHLH1*, was cloned and the encoded protein was characterized; the role of the *PnbHLH1* transcription factor was analyzed in the biosynthesis of PNS which might provide a theoretical and scientific basis for the efficient regulation of PNS biosynthesis and establish homologous or heterologous expression systems of saponins in *P. notoginseng*.

## Figures and Tables

**Table 1:** Sequences of the primers used in this study.

Primer Name	Primer Sequence (5′–3′)
HptⅡ-F	GAAGTGCTTGACATTGGGGAAT
HptⅡ-R	AGATGTTGGCGACCTCGTATT
18S rRNA-F	AACCATAAACGATGCCGACCAG
18S rRNA-R	TTCAGCCTTGCGACCATACTCC
PnSS-F	GCAGGACTTGTTGGATTAGGGT
PnSS-R	AACATGCGTGACTTTGGTATCTC
PnHMGR-F	GGCAGGACCCAGCACAAAATA
PnHMGR-R	ACACCCAGAAGGTTCAAGCAA
PnFPS-F	CGGATGCTGGACTATAATGTG
PnFPS-R	ATTTACGGCAATCATACCAACC
PnDS-F	TTTGGGAGCCTCCAGTTCCAAAACC
PnDS-R	TCTCTTTCTGACGATGCCCAGGATG
PnSE-F	TCTCCATGACTCATCAACCCTC
PnSE-R	CGGCCAACGCCATAGATAG
PnCAS-F	CGGATGGTTTATCTGCCTATGTC
PnCAS-R	GGTTGCGTGCTTGATTCCA
PnbHLH1-F	ACCTATTGATCTTATGACCTCCCA
PnbHLH1-R	CATCTTCTTTAGGCCAGGGA

**Table 2:** The probe sequences contained the E-box core sequences or mutation sequences.

Primer	Primer Sequence (5′–3′)
E-box-F	AATGCANNTGTTGGGGAGCANNTGTTGGGGAGCANNTGGGGA
E-box-R	TCCCCANNTGCTCCCCAACANNTGCTCCCCAACANNTGCATT
mE-box-F	AATGATTATTTTGGGGAGATTATTTTGGGGAGATTATTGGGA
mE-box-R	TCCCAATAATCTCCCCAAAATAATCTCCCCAAAATAATCATT
